# Robotic versus laparoscopic right hemicolectomy: a systematic review of the evidence

**DOI:** 10.1007/s11701-024-01862-5

**Published:** 2024-03-11

**Authors:** Jeremy Meyer, Elin Meyer, Guillaume Meurette, Emilie Liot, Christian Toso, Frédéric Ris

**Affiliations:** 1 Division of Digestive Surgery, University Hospitals of Geneva, Rue Gabrielle-Perret-Gentil,14, 41211 Geneva, Switzerland; 2 Medical School, University of Geneva, Rue Michel-Servet, 11206 Geneva, Switzerland; 3 Karolinska Institutet, Solnavägen 1, 171 77 Stockholm, Sweden

**Keywords:** Robotic surgery, MIS, RHC, Colorectal cancer, Colon cancer, Da Vinci

## Abstract

Robotics may facilitate the realization of fully minimally invasive right hemicolectomy, including intra-corporeal anastomosis and off-midline extraction, when compared to laparoscopy. Our aim was to compare laparoscopic right hemicolectomy with robotic right hemicolectomy in terms of peri-operative outcomes. MEDLINE was searched for original studies comparing laparoscopic right hemicolectomy with robotic right hemicolectomy in terms of peri-operative outcomes. The systematic review complied with the PRISMA 2020 recommendations. Variables related to patients’ demographics, surgical procedures, post-operative recovery and pathological outcomes were collected and qualitatively assessed. Two-hundred and ninety-three publications were screened, 277 were excluded and 16 were retained for qualitative analysis. The majority of included studies were observational and of limited sample size. When the type of anastomosis was left at surgeon’s discretion, intra-corporeal anastomosis was favoured in robotic right hemicolectomy (4/4 studies). When compared to laparoscopy, robotics allowed harvesting more lymph nodes (4/15 studies), a lower conversion rate to open surgery (5/14 studies), a shorter time to faeces (2/3 studies) and a shorter length of stay (5/14 studies), at the cost of a longer operative time (13/14 studies). Systematic review of existing studies, which are mostly non-randomized, suggests that robotic surgery may facilitate fully minimally invasive right hemicolectomy, including intra-corporeal anastomosis, and offer improved post-operative recovery.

## Introduction

Based on the results of the COLOR, UK MRC CLASICC and EnROL trials [1–5], professional societies recommend, whenever possible, choosing a minimally invasive approach over an open approach for performing right hemicolectomy [3, 4]. Nowadays, laparoscopy has become the standard approach for performing minimally invasive right hemicolectomy, representing 90.5% of minimally invasive right hemicolectomy in Denmark [6], 96.2% in the USA [7] and in 97.1% in Australia [8]. However, laparoscopy faces several technical limitations inherent to the technique, which have consequences for patients requiring right hemicolectomy.

For instance, central lymphadenectomy at the level of the middle colic artery is technically challenging using laparoscopic instruments and may have to be performed through the extraction site [9], which is sometimes referred to as laparoscopic-assisted right hemicolectomy [10]. Moreover, laparoscopy may lead to inadequate lymphadenectomy at the level of the ileo-colic pedicle itself, as laparoscopic instruments are not articulated and do not allow carefully dissecting around vessels close to its origin (central vascular ligation). Suboptimal lymphadenectomy may have consequences on patients’ survival, as some patients may be understaged and may not benefit from adjuvant chemotherapy. As a postulated consequence, analysis of the Danish Colorectal Cancer Group database for years 2008–2011 reported suboptimal 4-year disease-free survival for TNM stages I and II right-sided colorectal cancer when surgery was performed using laparoscopy [11], which led to the development of complete mesocolic excision (CME) as a correction measure.

Moreover, analysis of the Danish Colorectal Cancer Group database for years 2014–2018 revealed that 93.6% of patients undergoing laparoscopic right hemicolectomy receive an extra-corporeal anastomosis [6]. This proportion was of 68.7% of patients in the MERCY study [12]. However, extra-corporeal anastomosis requires a midline incision and extended mobilization of the mesenteries of the two bowel ends to join together, which expose patients to increased risks of post-operative pain, surgical site infection and post-operative ileus, which can translate into longer length of stay than intra-corporeal anastomosis [10, 13–20]. The low proportion of patients who receive intra-corporeal anastomosis during minimally invasive right hemicolectomy may be explained by the increased technical difficulty of performing intra-corporeal anastomosis when compared to extra-corporeal anastomosis when using a laparoscopic approach. 

As a consequence of extra-corporeal anastomosis, analysis of retrospective cohort study from the Cleveland clinic revealed that the midline was chosen as an extraction site during laparoscopic right hemicolectomy in 88.7% of patients, whereas a C-section was only used in a minority of patients [21]. The preferential choice of midline as an extraction site during laparoscopic right hemicolectomy and/or extra-corporeal anastomosis was confirmed by other reports [14, 15, 22–24], and unfortunately exposes patients to increased risks of wound-related complications, notably incisional hernia. For instance, a systematic review and meta-analysis estimated the pooled incidence of incisional hernia at the extraction site after laparoscopic colorectal surgery to be of 10.6% when using the midline and 0.9% when using a C-section [25]. 

We believe that robotics could potentially contribute to generalize a fully minimally invasive surgery approach for right hemicolectomy, including realization of intra-corporeal anastomosis, and this could translate into better post-operative recovery outcomes when compared to the current standard of care, which is laparoscopic right hemicolectomy with extra-corporeal anastomosis. Robotic right hemicolectomy could shorten the recovery of bowel function and, indirectly, shorten the length of stay. Moreover, it could decrease the incidence of wound-related complications, including incisional hernia, by facilitating off-midline extraction site. This could lead to significant savings for healthcare systems which could balance the increased surgical procedure-related costs attributed to robotic right hemicolectomy.

Our objective was therefore to determine whether robotics improves intra-operative, post-operative outcomes and/or pathological outcomes of right hemicolectomy when compared to laparoscopy, or not.

## Methods

The systematic review of the literature was reported in line with the PRISMA 2020 (Preferred Reporting Items for Systematic Reviews and Meta-Analyses) recommendations [26] (Table S1). MEDLINE was searched without time limit to the 07.08.2023 for observational studies and randomized controlled trials written in English and comparing laparoscopic right hemicolectomy with robotic right hemicolectomy in terms of intra-operative outcomes (such as conversion rate) and/or post-operative outcomes (such as length of stay, time to first faeces, 30-day morbidity, 30-day mortality), as reported in Table 1. The search build is reported in Table 2. Additional records were identified by screening references from secondary analyses in the field.

Observational studies including less than 50 patients per group (laparoscopy or robotic), letters, congress abstracts and secondary analyses were excluded. Publications not reporting on intra-operative and/or post-operative outcomes were excluded. Medians or means for the main outcome variables were extracted from included publications and summarized in tables. If needed, raw number of patients were calculated from proportions. Two authors (JM, EM) performed the selection of studies and extracted the data. In case of disagreement, consensus was reached with a third author (FR). Considering the heterogeneity in terms of patients’ populations, interventions (including or not CME or D3 lymphadenectomy), as well as potential duplicate patients (several publications were based on the ACS-NSQIP database), meta-analysis was not performed. Institutional review board approval was not required.

**Table 1:** Methods for the systematic review

Population	Intervention	Control	Outcome(s)	Design
Colorectal cancer and/or benign disease	Robotic RHC	Laparoscopic RHC	Intra- and/or post-operative outcome(s)	Observational and/or RCT

RHC: right hemicolectomy; RCT: randomized controlled trial

**Table 2:** Literature search strategy

Source of data	Search build
Database: MEDLINE Date: 07.08.2023	((Right hemicolectomy[Title/Abstract]) OR (right colectomy[Title/Abstract]) OR (complete mesocolic excision[Title/Abstract])) AND (robotic[Title/Abstract])

## Results

### Inclusion process

**Fig. 1:**
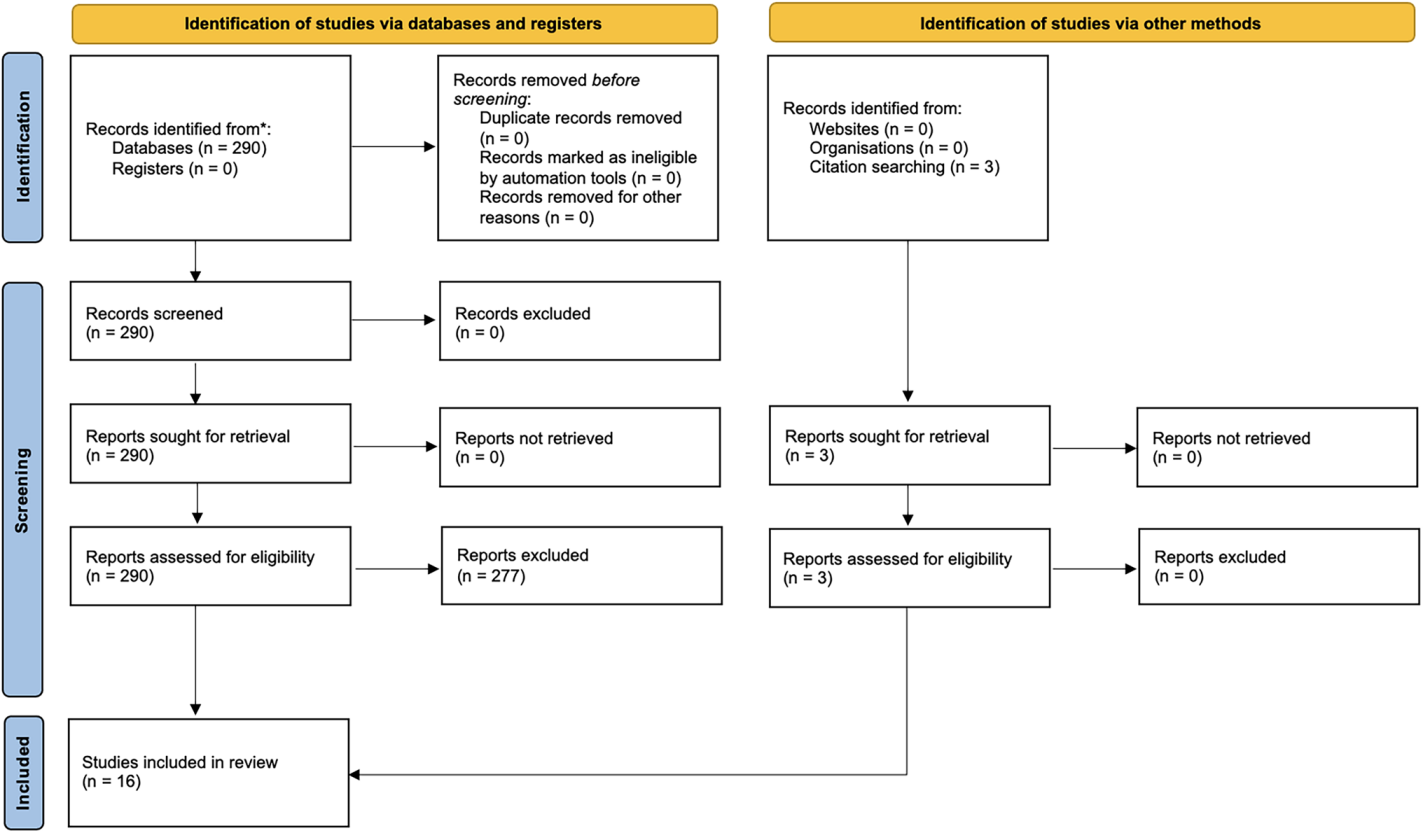
PRISMA flowchart

Two-hundred and ninety publications were identified on Medline. Additional records were identified from secondary analyses in the field. After application of inclusion and exclusion criteria, 16 publications [6, 8, 9, 27–39] were included in the qualitative analysis (Fig. 1).

### Characteristics of included studies

Fourteen publications were retrospective observational studies [6, 8, 9, 27–37]. Among them, three were propensity score-matched [27–29]. One study was prospective but non-randomized [39]. There was only one randomized controlled trial comparing robotic right hemicolectomy with laparoscopic right hemicolectomy [38]. The majority of studies were performed in the USA [9, 34–37] and in Italy [29–31, 33, 40]. Two studies, from Denmark [6] and Australia [8], used national databases to compare the outcomes of the surgical techniques. Interventions (robotic right hemicolectomy) and controls (laparoscopic right hemicolectomy) differed widely in terms of extent of lymphadenectomy (complete mesocolic excision or not, D3 lymphadenectomy or not), type of anastomosis (intra-corporeal or extra-corporeal) and extraction sites (midline or off-midline), as reported in Table 3. Of note, the MIRCAST study [39] compared robotic right hemicolectomy with either intra-corporeal anastomosis or extra-corporeal anastomosis with laparoscopic right hemicolectomy with either intra-corporeal anastomosis or extra-corporeal anastomosis. The number included patients ranged between 35 [38] to 6’521 patients [36] for the laparoscopic group, and between 35 [38] to 409 patients [39] for the robotic group.

**Table 3:** Characteristics of included studies

	Country	Setting	Data collection	Period	Population	Intervention	Robot	Control
Ruiz (2023) [39]	Spain	Multicentric	Prospective cohort	11.2018–11.2021	CRC or polyp	Robotic RHC with ICA/ECA	Da Vinci	Laparoscopic RHC with ICA/ECA
Tian (2023) [27]	China	Multicentric	Retrospective, PSM	07.2016–07.2021	CRC	Robotic RHC with CME, ECA	Da Vinci Si	Laparoscopic RHC with CME, ECA
De Angelis (2023) [28]	France	Multicentric	Retrospective, PSM	01.2014–12.2020	CRC	Robotic RHC ± CME, ICA	Da Vinci	Laparoscopic RHC ± CME, ICA
Clarke (2022) [8]	Australia	Nationwide	Retrospective cohort	01.2007–07.2020	CRC	Robotic RHC	–	Laparoscopic RHC
Dohrn (2021) [6]	Denmark	Nationwide	Retrospective cohort	01.2015–12.2018	CRC	Robotic RHC	–	Laparoscopic RHC
Merola (2020) [29]	Italy	Multicentric	Retrospective cohort, PSM	01.2012–08.2017	CRC	Robotic RHC	Da Vinci Si, Da Vinci Xi	Laparoscopic RHC
Tagliabue (2020) [30]	Italy	Monocentric	Retrospective cohort	01.2014–09.2019	CRC or polyp	Robotic RHC, ICA	Da Vinci	Laparoscopic RHC
Megevand (2019) [31]	Italy	Monocentric	Retrospective cohort	2010–2015	CRC	Robotic RHC without CME	Da Vinci	Laparoscopic RHC without CME
Solaini (2019) [32]	Italy	Multicentric	Retrospective cohort	02.2007–12.2017	–	Robotic RHC with ICA	–	Laparoscopic RHC with ICA
Spinoglio (2018) [33]	Italy	Monocentric	Retrospective cohort	10.2005–11.2013	CRC	Robotic RHC with CME, ICA	Da Vinci, Da Vinci Si	Laparoscopic RHC with CME, ICA
Haskins (2018) [34]	USA	Multicentric	Retrospective cohort	2012–2014	CRC	Robotic RHC	–	Laparoscopic RHC
Lujan (2018) [35]	USA	Multicentric	Retrospective cohort	01.2009–03.2015	CRC and benign	Robotic RHC with ICA	Da Vinci Si, Da Vinci Xi	Laparoscopic RHC with ECA
Widmar (2017) [9]	USA	Monocentric	Retrospective cohort	01.2012–12.2014	CRC	Robotic RHC	–	Laparoscopic RHC
Dolejs (2017) [36]	USA	Multicentric	Retrospective cohort	2012–2014	CRC and benign	Robotic RHC	–	Laparoscopic RHC
Casillas (2014) [37]	USA	Monocentric	Retrospective cohort	01.2005–04.2012	–	Robotic RHC with ECA	–	Laparoscopic RHC with ECA
Park (2012) [38]	South Korea	Monocentric	Randomized controlled trial	09.2009–07.2011	CRC	Robotic RHC with D3 lymphadenectomy	Da Vinci	Laparoscopic RHC with D3 lymphadenectomy

CRC: colorectal cancer; RHC: right hemicolectomy; ICA: intra-corporeal anastomosis; ECA: extra-corporeal anastomosis; PSM: propensity score-matched

**Table 4:**
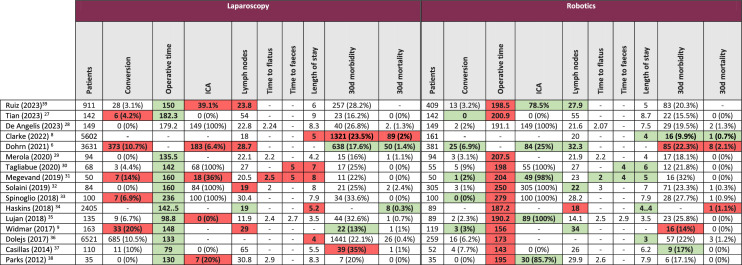
Outcomes of laparoscopic and robotic right hemicolectomy

*ICA* intra-corporeal anastomosis

In bold are indicated the variables found to be statistically different between groups according to the authors of included publications. In red are indicated the statistically significant differences in favor of the comparative group, and in green the variables in favor of the assessed group

### Per-operative outcomes

The operative time ranged between 79 min [37] and 182.3 min [27] for laparoscopic right hemicolectomy, and between 143 min [37] and 279 min [33] for robotic right hemicolectomy. Longer operative times were often reported in studies where complete mesocolic excision or D3 lymphadenectomy was performed. Thirteen studies found that operative time was statistically longer when a robotic approach was chosen over a laparoscopic approach [9, 27, 29–39]. Five studies reported that the conversion rate to open surgery was statistically lower with robotic right hemicolectomy than with laparoscopic right hemicolectomy [6, 9, 27, 31, 33], and no study found a lower conversion rate when using a laparoscopic approach. Some studies only included patients who underwent either extra-corporeal anastomosis or intra-corporeal anastomosis in both groups. Other studies compared robotic right hemicolectomy with intra-corporeal anastomosis to laparoscopic right hemicolectomy with extra-corporeal anastomosis [35], or robotic right hemicolectomy with either intra- or extra-corporeal anastomosis to laparoscopic right hemicolectomy with either intra- or extra-corporeal anastomosis [39]. A few studies did not pre-specify the type of anastomosis to perform and allowed comparing the effect of the surgical approach on the type of anastomosis chosen. When including only these studies [6, 31, 38, 39], intra-corporeal anastomosis was favored by surgeons when performing robotic right hemicolectomy, whereas extra-corporeal anastomosis was mostly performed during laparoscopic right hemicolectomy. Noteworthy, in the randomized controlled trial by Park et al. [38], the proportion of patients who had intra-corporeal anastomosis was of 85.7% in the robotic group, and of 20% in the laparoscopic group. Finally, probably due to the different levels of lymphadenectomy employed, the number of harvested lymph nodes varied widely among studies, and ranged from 11.9 [35] to 55 [27]. Four studies reported that the number of harvested lymph nodes was higher with the robotic approach [6, 9, 32, 39], and one study found that the number of harvested lymph nodes was higher with the laparoscopic approach [34](Table 4).

### Post-operative outcomes

Recovery of bowel function was estimated using the time to flatus, the time to faeces (first bowel opening) or a combination of both (time to recovery of bowel function). In terms of time to flatus, only one study found a statistically significant difference of -0.5 day between both surgical techniques in favour of robotics [31]. In terms of time to faeces, two studies reported a difference of -1 day between groups, again in favour of robotic right hemicolectomy [30, 31]. After right hemicolectomy, the length of stay ranged between 3 days [36] and 8 days [31]. The length of stay was reported by five studies to be significantly shorter after robotic right hemicolectomy than after laparoscopic right hemicolectomy [8, 30, 31, 34, 36]. No study reported a shorter length of stay in favour of laparoscopic right hemicolectomy. Post-operative morbidity after right hemicolectomy ranged between 9.9% [8] and 35% [37]. Post-operative morbidity was reported to be statistically different between surgical approaches in four studies [6, 8, 9, 37], with two studies [6, 9] favouring laparoscopy and two studies [8, 37] favouring robotics. Post-operative mortality was inferior to 2.4% [32] in all studies, and eight studies reported 0% mortality when performing robotic right hemicolectomy [9, 27, 29–31, 35, 37, 38] (Table 4).

## Discussion

After the publication of several trials showing that laparoscopy offered similar oncological outcomes for improved recovery outcomes for minimally invasive right hemicolectomy [1–5], laparoscopy has become the standard approach for performing the procedure [6–8]. Nowadays, laparoscopic right hemicolectomy consists mostly in laparoscopic mobilization of the colon, extra-corporeal vessel division and lymphadenectomy (at least at the level of the ileo-colic vessels), extra-corporeal anastomosis and extraction through the midline [6, 9, 12, 14, 15, 21–24]. However, fully minimally invasive right hemicolectomy should theoretically include intra-corporeal vessel division, lymphadenectomy and anastomosis, allowing to use a C-section as extraction site. Such a procedure is possible by laparoscopy, but not universally adopted, because of the technical limitations of laparoscopy. On this aspect, robotic platforms, which offer improved minimally invasive possibilities, may allow optimizing the technique for right hemicolectomy and reaching better post-operative outcomes.

In a systematic review of the literature in the field, we identified 16 publications comparing laparoscopic right hemicolectomy with robotic right hemicolectomy [6, 8, 9, 27–39]. These publications showed that patients who undergo robotic right hemicolectomy have decreased blood loss, decreased incidence of post-operative complications, shorter recovery of bowel function, fewer conversions to open surgery and shorter length of stay, at the cost of a slightly longer operative time when compared to laparoscopy [41–44]. The improved post-operative outcomes observed with robotics may be explained by the facilitated realization of intra-corporeal anastomosis when compared to laparoscopy. For instance, a few publications reported that the proportion of patients who received intra-corporeal anastomosis was increased with robotics when compared to laparoscopy [12, 21, 38, 39, 45]. In the only randomized controlled trial published in the field, the proportion of intra-corporeal anastomosis was of 85.7% in patients undergoing robotic right hemicolectomy versus 20% in patients undergoing laparoscopic right hemicolectomy [38]. As a corollary, a C-section may have been chosen as the preferential extraction site in these patients, as it is the case in 77.6% of patients (98.7% for off-midline extraction site) undergoing intra-corporeal anastomosis when compared to 0% of those undergoing extra-corporeal anastomosis [24]. Noteworthy, in the MIRCAST study, extraction through a C-section was more often done in patients who underwent intra-corporeal anastomosis (odds ratio: 165.7, *p* < 0.001) [39]. However, the vast majority of these publications were not devoid of potential bias, as they reported the results of observational studies, included heterogeneous populations of patients and compared heterogeneous surgical techniques including or not intra-corporeal anastomosis, D3 lymphadenectomy and/or complete mesocolic excision. For instance, D3 lymphadenectomy was more often performed in the robotic group in the MIRCAST study (odds ratio: 4.22, *p* < 0.001) [39], which may increase the operative time of the technique and prevent any objective comparison with laparoscopy. Moreover, the majority of included studies were of limited sample sizes, were based on database analysis and/or on non-randomized data. Therefore, high-quality randomized evidence is needed to validate the findings of these early observational studies.

To date, only one randomized controlled trial compared robotic right hemicolectomy with laparoscopic right hemicolectomy [38]. In this trial, no difference was found in terms of time to first passage of flatus, length of stay, complications, postoperative pain and number of harvested lymph nodes between the two surgical techniques. Long-term analysis of the data showed similar long-term survival between the two techniques [46]. However, one main limitation of this trial was represented by the fact that the anastomosis technique was not standardized between the surgical approaches: patients who underwent robotic right hemicolectomy could receive either intra-corporeal or extra-corporeal anastomosis. Therefore, the potential main advantage of the robotic approach, which is intra-corporeal anastomosis [44], was not properly evaluated. It should, however, be noted that intra-corporeal anastomosis was more often performed in patients who underwent robotic right hemicolectomy than in patients who had laparoscopic right hemicolectomy, meaning that robotic right hemicolectomy facilitated the realization of intra-corporeal anastomosis. This assertion was confirmed by a systematic review and meta-analysis [44]. Moreover, the trial was powered on the length of stay, which was longer than nowadays standards and longer than our own personal experience with robotic right hemicolectomy, which is of 5.4 ± 3.8 days (unpublished data), and therefore, limits the validity of its findings. Finally, the total sample size was of 70 patients, which may have been insufficient to show a potential difference between groups for several of the assessed variables (type II statistical error).

From a personal point of view, we believe that the true benefits of robotic right hemicolectomy can only be only achieved when opting for a fully minimally invasive robotic approach including intra-corporeal anastomosis. A propensity score-matched analysis of 192 patients who underwent either totally robotic right hemicolectomy (including intra-corporeal anastomosis) or robotic-assisted right hemicolectomy (including extra-corporeal anastomosis) showed that improved post-operative outcomes were reported when using the totally robotic approach, in terms of post-operative pain and recovery of bowel function [47]. In a prospective cohort study of totally robotic right hemicolectomy [48], we showed that intra-corporeal anastomosis was achievable in all patients, with a low conversion rate of 3.3% and a reasonable mean operative time of 200.4 ± 114.9 min (which has to be compared to operative times up to 279 min [33] and 327.5 min [49] documented in the literature). Moreover, we reported a mean number of harvested lymph nodes of 22.4 ± 7.6, a mean length of stay was of 5.4 ± 3.8 days, a post-operative morbidity of 11.7% and no mortality. However, we note that a national audit showed that intra-corporeal anastomosis is still not routinely performed during robotic right hemicolectomy [6]. Moreover, this approach has to be compared to the actual standard of care, which is laparoscopic right hemicolectomy with extra-corporeal anastomosis, and additional longer term outcomes remain to be evaluated, such as the incidence of incisional hernia (which is modulated by the choice of extraction site). The experience of the operating surgeon on a robotic platform should also be sufficient, as it was shown that the operative time and the incidence of conversion to open surgery were inversely correlated to the personal caseload [50]. To conclude, systematic review of existing studies, which are mostly non-randomized, suggest that robotic surgery may offer improved post-operative outcomes after right hemicolectomy when compared to laparoscopic surgery, notably by facilitating the realization of intra-corporeal anastomosis and off-midline extraction of the operative specimen, as well as extended lymph node dissection. Results of ongoing randomized controlled, such as the PRORHEM trial or the ROLACART-1 pilot trial are awaited for confirming these results.

## Data Availability

No datasets were generated or analysed during the current study.
